# The concurrence of the current postanalytical phase management with the national recommendations: a survey of the Working Group for Postanalytics of the Croatian Society of Medical Biochemistry and Laboratory Medicine

**DOI:** 10.11613/BM.2021.030704

**Published:** 2021-10-15

**Authors:** Anja Jokic, Vladimira Rimac, Jelena Vlasic Tanaskovic, Sonja Podolar, Lorena Honovic, Jasna Lenicek Krleza

**Affiliations:** 1 Department of Medical Biochemistry, Hematology and Coagulation with Cytology, University Hospital for Infectious Diseases “Dr. Fran Mihaljević”, Zagreb, Croatia; 2 Working Group for Post-analytics, Croatian Society of Medical Biochemistry and Laboratory Medicine, Zagreb, Croatia; 3 Department of Transfusion Medicine and Transplantation Biology, University Hospital Centre Zagreb, Zagreb, Croatia; 4 Department of Laboratory Diagnostics, General Hospital Pula, Pula, Croatia; 5 Croatian Centre for Quality Assessment in Laboratory Medicine, Croatian Society of Medical Biochemistry and Laboratory Medicine, Zagreb, Croatia; 6 Medical Biochemistry Laboratory, General Hospital “Dr. Tomislav Bardek”, Koprivnica, Croatia; 7 Department of Laboratory Diagnostics, Children’s Hospital Zagreb, Zagreb, Croatia

**Keywords:** postanalytical phase, survey, clinical laboratory, critical results, quality indicators

## Abstract

**Introduction:**

The detection and prevention of errors in the postanalytical phase can be done through the harmonization and standardization of constituent parts of this phase of laboratory work. The aim was to investigate how well the ongoing management of the postanalytical phase corresponds to the document “Post-analytical laboratory work: national recommendations” in Croatian medical biochemistry laboratories (MBLs).

**Materials and methods:**

All 195 MBLs participating in the national external quality assessment scheme, were invited to undertake a part in a survey. Through 23 questions the participants were asked about management of the reference intervals (RI), delta check, reflex/reflective testing, postanalytical quality indicators and other parts of the postanalytical phase recommended in the national recommendations. The results are presented in numbers and percentages.

**Results:**

Out of 195 MBLs, 119 participated in the survey, giving a response rate of 61%. Not all of the respondents provided answers to all the questions. Delta check has not been used in 59% (70/118) of the laboratories. Only 22/113 (20%) laboratories use reflex and/or reflective testing. In 53% of the laboratories, critical results were reported within 30 minutes of the confirmation of the results. In 34% (40/118) of the laboratories, turnaround time and reporting of critical results are two most often monitored postanalytical quality indicators.

**Conclusion:**

The results showed the critical results reporting and monitoring of postanalytical quality indicators are in the line with the recommendations. However, the management of RI verification, the use of delta check and reflex/reflective testing still must be harmonized among Croatian MBLs.

## Introduction

In the past few decades, informatization has contributed to the improvement of the total testing process, and it has carried out a significant role in minimizing errors in the postanalytical phase. Informatization enables the effective release of laboratory test results, turnaround time (TAT) monitoring, and improves the timeliness of notification of critical results. Despite that, 13-20% of errors in the total testing process are still postanalytical errors (*1*, *2*).

Defining protocols to be followed in the postanalytical phase of laboratory work is necessary to reduce errors, but also to facilitate the harmonization of this ultimate step in laboratory testing. To improve the postanalytical phase of laboratory work, each laboratory must define quality indicators to be monitored, one of the fundamental requirements for the accreditation of a clinical laboratory (*3*, *4*). The process of monitoring and improving the key components of the postanalytical phase goes beyond the TAT and critical result monitoring, the usual quality indicators followed by the laboratories. It should also address the use of delta check, repeat testing, reference intervals (RIs), and interpretative comment (*5*, *6*).

Delta check has been proven to be a good tool for improving the postanalytical as well as preanalytical phase. It is especially significant for the results falling within the critical limits defined by the laboratory. Repeating measurements for results within the critical limits remain the standard practice in many laboratories, despite the recommendations that the results without the flag coming from the analyser with accepted quality control are unnecessary. Recognition, documentation, and notification of critical results, as well as prompt communication to a physician, are crucial for patient safety (*3*, *5*, *7*).

Each laboratory test report must contain units of measurement, followed by appropriate RI for the specific age-group. In addition to the RIs on the laboratory test report, an interpretative comment may also be important for physicians because it facilitate the interpretation of test results (*7*). Turnaround time is the most common quality indicator of the postanalytical phase. It is significant to emphasize that delays in any phase of laboratory work may lead to an increased TAT (*e.g.*, repeat testing or preanalytical errors like sample clot or haemolysed sample) (*5*, *8*).

In 2019 the Working Group for Postanalytics of the Croatian Society of Medical Biochemistry and Laboratory Medicine (CSMBLM) published the document “Post-analytical laboratory work: national recommendations” for the postanalytical phase of laboratory work covering the main topics such as the evaluation of test results, the release of the laboratory test report, sample storage and disposal, the archiving of laboratory documentation, and managing postanalytical quality indicators. The recommendations were issued in an attempt to contribute to the harmonization of the postanalytical phase of laboratory work in Croatia (*7*).

This study represents the first comprehensive evaluation of the management of some key elements of the postanalytical phase of laboratory work in Croatian medical biochemistry laboratories (MBLs) after the national recommendations has been published. Recognising that the implementation of national recommendations is not obligatory, their utilization and implementation can be considered good laboratory practice and the improvement of laboratory work.

The study aimed to investigate how well the ongoing management of the postanalytical phase in Croatian MBLs corresponds to the document “Post-analytical laboratory work: national recommendations”.

## Materials and methods

### Study design

A study in the form of a survey was conducted by the Working Group for Postanalytics in collaboration with the Croatian Centre for Quality Assessment in Laboratory Medicine, both within the CSMBLM. An online invitation was extended in September 2019 to all 195 MBLs in Croatia. Laboratories’ participation in the survey was optional and voluntary. All the respondents to the survey were taken into consideration and the laboratories that did not answer the particular question were not excluded.

The survey was part of Module 10 entitled “Postanalytical phase of laboratory testing” and conducted as a part of the national external quality assessment scheme. It contained 23 questions and laboratories’ anonymity was assured through code numbers which were selected by the participants. The questionnaire had been designed according to the document “Post-analytical laboratory work: national recommendations”. The questions (yes/no and multiple-choice questions) in the survey assessed the laboratory management of the key elements of the postanalytical phase; RI, delta check, reflex and/or reflective testing, reporting of critical results, sample storage, and postanalytical quality indicators.

### Data analysis

The results were obtained by counting and are presented in numbers and percentages. The percentage was calculated according to the total number of answers to the particular question. The concordance was characterized as satisfactory when more than half of the answers to a particular question were in the line with the national recommendations. The data analysis was carried out using Microsoft Excel version 2016 (Microsoft, Redmond, USA).

## Results

Out of 195 MBLs in Croatia, 119 participated in the survey, giving a response rate of 61%. However, not all of the respondents provided answers to all the questions. Overall survey results are presented in Table 1.

**Table 1:** Distribution of participants’ answers regarding the management of the postanalytical phase among Croatian laboratories

**Question and answer**	**n/N, (%)**
**1. What type of health care setting does your laboratory belong to?**	
A. Primary health care	78/119 (66)
B. Secondary health care	30/119 (25)
C. Tertiary health care	11/119 (9)
**2. When there is no reference interval recommended by the Croatian Chamber of Medical Biochemists (CCMB), which other source do you use for the reference interval?**	
A. Reference interval declared by the reagent manufacturer	91/115 (79)
B. Reference interval as a result of multi-centric studies	1/115 (1)
C. Reference interval from literature and the current state-of-the-art	23/115 (20)
**3. Have you verified the acceptability of the applied reference interval in your laboratory?**	
A. Yes	42/113 (37)
B. No (Please give a reason)	71/113 (63)
**4. What do you do when there is no reference interval for a particular age group of patients?**	
A. We combine with the next age group and issue a result with a reference interval of that age group	53/114 (46)
B. We issue the result without the corresponding reference interval with an explanation in the “Comments” area on the laboratory test report	42/114 (37)
C. Other, please specify	19/114 (17)
**5. Do you have a delta check (testing the difference between two consecutive results for the same patient) implemented in the laboratory information system (LIS)?**	
A. Yes	21/118 (18)
B. No, we use the manual calculation method	27/118 (23)
C. We do not use delta check at all	70/118 (59)
6. If the answer to the previous question is Yes, how did you define “delta check” in LIS? *	
A. Reference change value	6/21
B. Delta difference	3/21
C. Delta percent change	10/21
D. Rate difference	0/21
E. Rate percent change	2/21
**7. For which areas of Clinical Chemistry do you use delta check (more answers possible)?**	
A. General biochemistry	27/86 (31)
B. Haematology	26/86 (30)
C. Coagulation	21/86 (24)
D. Special biochemistry	15/86 (17)
E. Emergency laboratory test	13/86 (15)
F. Other, please specify	2/86 (2)
G. We do not use it	52/86 (61)
8. Which is the timeframe of delta check in your laboratory? *	
A. 1-3 days	4/32
B. 2-5 days	5/32
C. > 5 days	23/32
**9. How do you handle a sample when diluting it manually?**	
A. According to the manufacturer’s instructions	76/118 (65)
B. According to our protocol	12/118 (10)
C. We do not dilute manually; we only use automatic dilution from the analyser	30/118 (25)
**10. What do you consider the most justifiable reason to repeat the measurement (more answers possible)?**	
A. A result accompanied by a remark from the analyser, whether inside or outside the measuring range	95/119 (80)
B. A result that has no remark from the analyser but is outside the measuring range	77/119 (65)
C. A result that is discrepant with other results	91/119 (77)
D. A result that is inconsistent with the patient’s diagnosis	73/119 (61)
**11. Do you use reflex and/or reflective testing in your laboratory?**	
A. Yes (Please specify)	22/113 (20)
B. No	91/113 (80)
**12. A physician requested total and direct bilirubin. The value of total bilirubin is within the reference interval. What would you do?**	
A. Regardless of the value of total bilirubin, direct bilirubin was determined because the physician asked for it	75/95 (79)
B. First, determine total bilirubin and based on the obtained value proceed according to the laboratory protocol on reflex testing	20/95 (21)
**13. What is your primary source of the critical results?**	
A. The CCMB document “Critical Laboratory Findings and Critical Result Reporting”	119/119 (100)
B. International professional organizations (the EFLM, the IFCC)	0/119 (0)
C. Other literary sources	0/119 (0)
**14. Do you report a critical result as soon as you get it or after a repeated testing?**	
A. Yes, immediately	21/119 (18)
B. Sometimes	10/119 (8)
C. After repeated measurement	88/119 (74)
**15. In what period do you report a critical result?**	
A. Within 30 minutes of the confirmation of results	62/118 (53)
B. Within one hour of the confirmation of results	9/118 (7)
C. When we manage to get a physician/department	47/118(40)
**16. Do you keep a record of the reported critical results?**	
A. Yes	99/118 (84)
B. Sometimes	7/118 (6)
C. No	12/118 (10)
**17. Do you use the recommended standardized remarks in the “Comments” area of the laboratory test report?**	
A. Yes, whenever possible	79/119 (66)
B. Sometimes	13/119 (11)
C. No, we have our own notes	27/119 (23)
**18. If the obtained test result requires additional interpretation, what do you do?**	
A. We notify the requesting physician/department	37/119 (31)
B. We explain in the “Comments” area on the laboratory test report	15/119 (13)
C. We explain in the “Comments” area on the laboratory test report and personally notify the requesting physician/department	64/119 (54)
D. We do not have such cases	3/119 (2)
**19. How long do you keep the samples after analysis?**	
A. 24 hours	41/119 (35)
B. 48 hours	36/119 (30)
C. > 48 hours	8/119 (7)
D. We throw them away at the end of the workday due to lack of adequate storage space	3/119 (2)
E. As long as it is recommended for each type of sample/analysis	31/119 (26)
**20. Which postanalytical phase quality indicators do you monitor in your laboratory?**	
A. Turnaround time (TAT)	40/118 (34)
B. Percentage of incorrect (revoked) laboratory test reports	6/118 (5)
C. Reporting critical results	40/118 (34)
D. We do not monitor them	32/118 (27)
**21. How do you monitor TAT in your laboratory?**	
A. From the time of registration of the request in the LIS to the time of confirmation/validation of the test result	52/118 (44)
B. From the time of sampling to the time of confirmation/validation of the test result	16/118 (14)
C. We do not monitor TAT	50/118 (42)
**22. In what form do you most often issue a laboratory test report?**	
A. On paper, to patients who come to the laboratory	9/118 (8)
B. On paper, to physician/wards	11/118 (9)
C. Electronically, via Hospital information system	98/118 (83)
**23. Do you send patients their laboratory test reports by e-mail?**	
A. Yes	21/119 (18)
B. Yes, but they must first file a request to send the laboratory test report by e-mail	48/119 (40)
C. No	50/119 (42)
Results are presented as the number of answers to each offered question option (n) and the percentage (%) of the total number of answers (N) to a particular question. *Due to the small sample size results are not presented with the percentage. IVD – *in vitro* diagnostic. CCMB – Croatian Chamber of Medical Biochemists. EFLM – European Federation of Clinical Chemistry and Laboratory Medicine. IFCC – International Federation of Clinical Chemistry and Laboratory Medicine.	

In the absence of RI recommended by the Croatian Chamber of Medical Biochemists (CCMB), 79% of the participating laboratories will use a RI declared by the *in vitro* diagnostics (IVD) manufacturer. Results showed that 63% (71/113) of the respondents had not conducted a verification of the acceptability of the applied RI in their laboratory (Table 1). The reasons for such practice were provided by 46 out of 71 of the participating laboratories: 15/46 of them mentioned economic reasons and/or lack of personnel for such practice, while the inability to gather enough representative samples was the reason pointed out by 11/46 of participating laboratories. The majority of laboratories, 20/46, considers the RIs issued by the CCMB reliable enough.

When asked about the management of laboratory reports in the absence of RI for a particular age group, only 17% (19/114) of the laboratories answered, stating the following reasons: some had not encountered such a situation (11/19), and some use the RI from the literature (8/19). In such cases, the result is accompanied by the explanation in the “Comments” area on the laboratory report. Delta check has not been used in the majority of the participating laboratories (59%) and among those that use it, only 21 laboratories implemented delta check in the LIS. Furthermore, those laboratories that use delta check, reported delta percent check and reference change value as the two most used methods of delta check in the LIS. More than two-thirds of the laboratories (72%) have set their timeframe of delta check longer than five days.

Only 22 out of 113 (20%) laboratories use reflex and/or reflective testing in their work. It is most often used in the field of biochemistry (15/22) and least often in coagulation (1/22). 74% of the laboratories will report critical result after repeating the analysis; 53% of the laboratories would do it within 30 minutes of the confirmation of critical result and 84% keeps a record of the critical result reporting (Table 1).

A total of 86 out of 118 laboratories (73%) monitor some type of quality indicators of the postanalytical phase, mostly TAT (34%) and the notification of critical results (34%) (Table 1).

## Discussion

The results reveal variations among Croatian laboratories in the receptivity towards the procedures to be followed in the postanalytical phase.

As defined by the national recommendations, laboratory test results should not be issued without corresponding RI for each analyte and the practice to be followed in the absence of RI has also been defined (*7*, *9*). Survey results revealed that the practice of all MBLs is concordant with the national recommendations in the nonexistence of RI recommended by the CCMB; other sources of RI have been chosen. In such cases, most Croatian laboratories would choose an RI declared by the IVD manufacturer, demonstrating an understanding of the importance of the RI that accompanies the result issued by the laboratory (*10*). Presumably, the accessibility of RI coming from the IVD manufacturer and their supposed reliability (because of legal requirements for the manufacturers in providing suitable RI) are the reasons for laboratories not to proceed with the extensive work in providing their own RI for a stated analyte (*11*).

One of the requirements of the International Organization for Standardization ISO 15189:2012 and national recommendations is that the laboratory periodically evaluates RIs (*4*, *7*). Most of the surveyed Croatian laboratories have not performed such evaluation. The obstacles for the verification of the RI mentioned by the laboratories in this survey (*e.g.*, availability of referent population and time-consuming process) are the same ones discussed by several prominent laboratory experts (*12*). Establishing own RI is even more challenging work for many reasons, such as selecting the representative population (*e.g.*, “healthy” geriatric population), harmonizing the preanalytical phase (*e.g.*, fasting *vs* non-fasting), and standardizing the analytical phase (*e.g.*, methods, calibrators) (*13*). The determination of RIs for the paediatric population places several other obstacles in front of the laboratory in terms of recruiting an adequate number of healthy children in each age-group, collecting sufficient blood volume, and defining RIs for the biomarkers showing alternations associated with the child’s growth (*e.g.*, alkaline phosphatase) (*14*). In such cases, one of the solutions could be a verification of RIs from the pre-existing paediatric database as it was shown in the study by Zrinski Topic *et al.* or conducting a posteriori study (*15*, *16*).

A relatively low concordance to the national recommendations regarding the use of delta check can be attributed to the fact that most laboratories were from primary health care settings. In those laboratories, the period between consecutive test results for the same patient is ordinarily more than the recommended 2-5 days, which makes the use of a delta check in primary health care laboratories questionable (*7*). Studies conducted in different health care systems and settings show the benefits of implementing delta check in daily work regarding, *e.g.*, detection of specimen mix-up errors, contaminated specimen (*e.g.*, IV fluid), and analytical error (*17*, *18*).

In a contemporary laboratory, reflex and/or reflective testing can add a new dimension to the purely analytical service of obtaining and issuing test results. Studies have shown that both physicians and patients view reflective testing favourably because it can assist in the process of establishing the patient diagnosis (*19*). Nonetheless, according to our results, most Croatian laboratories do not use reflex and/or reflective testing. The plausible reason is that most of the surveyed laboratories were from the primary health care settings, where organisation of primary health care settings on the national level disables a laboratory from conducting additional testing before consulting with a requesting physician. The International Federation of Clinical Chemistry and Laboratory Medicine considers reporting of critical results as one of the important indicators of the postanalytical phase quality control (*20*). Most of the surveyed Croatian laboratories report critical results after repeat testing and half of them to report it within 30 minutes of the confirmation of the results, as recommended (*7*). The College of American Pathologists in its Q-Probes survey reported 61% of the laboratories retest critical chemistry results (*21*). In the Portuguese study, 60% of the laboratories notify critical results within 15 minutes and 33% within 30 minutes of the timeframe. Similar results are documented in the Croatian study; 63% of the laboratories report the critical result within 15 minutes and 28% within 30 minutes after the measurement (*22*, *23*).

Incorrect laboratory reports, TAT, and the notification of critical results are three recommended quality indicators for the postanalytical phase (*5*, *7*). Our results show one-third of the laboratories do not meet these minimum standards. One of the reasons might be that most of our responders come from the primary health care settings, where the laboratory work is organized in a way that the results are issued after the routine work is done, or even the next working day. Such organization diminishes the need for the prompt laboratory results release. In the survey conducted by Preston, 33% of the laboratories monitor TAT, which is remarkably similar to our results (34%). However, the before mentioned study reported 62% of the laboratories monitoring notification of critical results, which is almost double what was reported in our study (*24*).

Our survey contains some limitations. We had not conducted questionnaire validation, which was potentially reflected on the quality of the survey. The time between the publication of the national recommendations and the conducted survey was three months and arguably this time was not long enough for the laboratories to implement recommendations thoroughly. That may be one of the reasons for the weaker implementation of the recommendations in some parts of the postanalytical phase. Furthermore, the number of participating laboratories from the secondary and tertiary health care settings was relatively limited, which contributes to not possessing much more insight into their management of the postanalytical phase. In the previous study conducted shortly after the publication of the national recommendations, autovalidation was surveyed (*25*). For that reason, autovalidation was not included in this survey.

In conclusion, this study was the attempt to investigate the agreement of laboratories’ management of postanalytical phase to the national recommendations. The issues with low concordance are RI verification, the use of delta check, and the reflex/reflective testing since more than half of reported results differ from the national recommendations. At the same time, critical results reporting and monitoring of the postanalytical quality indicators indicate satisfactory concordance with the national recommendations. Although the results revealed that the overall postanalytical phase management has room for improvement, there is a promising basis for the future harmonization and standardization of the postanalytical phase in Croatian MBLs.

## References

[1] Plebani M Laposata M Lundberg GD. The brain-to-brain loop concept for laboratory testing 40 years after its introduction. Am J Clin Pathol. 2011;136:829–33. 10.1309/AJCPR28HWHSSDNON 22095366

[2] Plebani M. The detection and prevention of errors in laboratory medicine. Ann Clin Biochem. 2010;47:101–10. 10.1258/acb.2009.009222 19952034

[3] Plebani M. Harmonization in laboratory medicine: requests, samples, measurements and reports. Crit Rev Clin Lab Sci. 2016;53:184–96. 10.3109/10408363.2015.1116851 26667798

[4] ISO International Organization for Standardization (ISO). EN ISO 15189 - Medical laboratories - Requirements for quality and competence. 3rd ed. Geneva, Switzerland: ISO; 2012.

[5] Zemlin AE. Errors in the Extra-analytical Phases of Clinical Chemistry Laboratory Testing. Indian J Clin Biochem. 2018;33:154–62. 10.1007/s12291-017-0657-2 29651205 PMC5891449

[6] Plebani M. Towards a new paradigm in laboratory medicine: the five rights. Clin Chem Lab Med. 2016;54:1881–91. 10.1515/cclm-2016-0848 27732557

[7] Lenicek Krleza J Honovic L Vlasic Tanaskovic J Podolar S Rimac V Jokic A. Post-analytical laboratory work: national recommendations from the Working Group for Post-analytics on behalf of the Croatian Society of Medical Biochemistry and Laboratory Medicine. Biochem Med (Zagreb). 2019;29:020502. 10.11613/BM.2019.020502 31223256 PMC6559616

[8] Sciacovelli L Aita A Padoan A Pelloso M Antonelli A Piva E Performance Criteria and Quality Indicators for the Post-Analytical Phase. Clin Chem Lab Med. 2016;54:1169–76. 10.1515/cclm-2015-0897 26656613

[9] Croatian Chamber of Medical Biochemists. [Preporuka za postupanje u nedostatku referentnog intervala Povjerenstva za stručna pitanja]. Available from: http://www.hkmb.hr/dokumenta/arhiva/arhivirane-obavijesti-2010/. Accessed 15 February 2018. (in Croatian)

[10] Ozarda Y. Reference intervals: current status, recent developments and future considerations. Biochem Med (Zagreb). 2016;26:5–16. 10.11613/BM.2016.001 26981015 PMC4783089

[11] Directive 98/79/EC of European Parliamant and the Council of 27 October 1998 on in vitro diagnostic medical devices. Offical J Eur Commun. 1998;L331/1-L331/37.

[12] Miller WG Horowitz GL Ceriotti F Fleming JK Greenberg N Katayev A Reference Intervals: Strengths, Weaknesses, and Challenges. Clin Chem. 2016;62:916–23. 10.1373/clinchem.2016.256511 27230874

[13] Ceriotti F Hinzmann R Panteghini M. Reference intervals: the way forward. Ann Clin Biochem. 2009;46:8–17. 10.1258/acb.2008.008170 19103955

[14] Carlin J Ignjatovic V Monagle P. Paediatric Reference Intervals: Current Status, Gaps, Challenges and Future Considerations. Clin Biochem Rev. 2020;41:43–52. 10.33176/AACB-19-00036 32518426 PMC7255313

[15] Zrinski Topić R Leniček Krleža J. Verification of the Canadian Laboratory Initiative on Paediatric Reference Intervals (CALIPER) reference values in Croatian children and adolescents. Biochem Med (Zagreb). 2020;30:020710. 10.11613/BM.2020.020710 32550818 PMC7271758

[16] Katayev A Balciza C Seccombe DW. Establishing Reference Intervals for Clinical Laboratory Test Results. Am J Clin Pathol. 2010;133:180–6. 10.1309/AJCPN5BMTSF1CDYP 20093226

[17] Ovens K Naugler C. How useful are delta checks in the 21st century? A stochastic-dynamic model of specimen mix-up and detection. J Pathol Inform. 2012;3:5. 10.4103/2153-3539.93402 22439125 PMC3307229

[18] He S Kang F Wang W Chen B Wang Z. National survey on delta checks in clinical laboratories in China. Clin Chem Lab Med. 2020;58:569–76. 10.1515/cclm-2019-1131 31927514

[19] Verboeket-van de Venne WP Aakre K Watine J Oosterhuis W. Reflective testing: adding value to laboratory testing. Clin Chem Lab Med. 2012;50:1249–52. 10.1515/cclm-2011-0611 22850057

[20] Sciacovelli L Lippi G Sumarac Z West J del Pino Castro IG Furtado Vieira K Working Group. “Laboratory Errors and Patient Safety” of International Federation of Clinical Chemistry and Laboratory Medicine (IFCC). Quality Indicators in Laboratory Medicine: the status of the progress of IFCC Working Group “Laboratory Errors and Patient Safety” project. Clin Chem Lab Med. 2017;55:348–57. 10.1515/cclm-2016-0929 27988505

[21] Paxton A. Critical value repeats: redundancy, necessity? CAP Today. 2010;24:1.

[22] Vuljanić D Pereira M Santos S Nikler A Biljak VR Cachapuz I. Critical Results Reporting in Portuguese Hospital Laboratories: State-of-the-Art. EJIFCC. 2020;31:145–56. 32549882 PMC7294818

[23] Kopcinovic LM Trifunovic J Pavosevic T Nikolac N. Croatian survey on critical results reporting. Biochem Med (Zagreb). 2015;25:193–202. 10.11613/BM.2015.019 26110031 PMC4470108

[24] Preston LJ. A survey of quality indicator use in the clinical laboratory. Clin Lab Sci. 2008;21:25–32. 18335858

[25] Rimac V Jokic A Podolar S Vlasic Tanaskovic J Honovic L Lenicek Krleza J. General position of Croatian medical biochemistry laboratories on autovalidation: survey of the Working Group for Post-analytics of the Croatian Society of Medical Biochemistry and Laboratory Medicine. Biochem Med (Zagreb). 2020;30:020702. 10.11613/BM.2020.020702 32292280 PMC7138006

